# The Medical Inspection and Treatment of School Children

**Published:** 1910-04-09

**Authors:** 


### The Hospital

A Journal of

The Medical Sciences and Hospital Administration.

New Series. No. 164, Vol. VII. [No. 1236, Vol. XLVIII.] SATURDAY, APRIL 9, 1910.

### The Medical Inspection and Treatment of School Children.

The magnitude of the duties cast upon local education authorities in respect of the medical inspection and treatment of school children, by an Act lightly passed in 1907, and loosely phrased, is beginning to engage serious consideration. The effects of legislation passed a generation ago making elementary education compulsory, with its corollary of making elementary education free, have, in the light of the awakening social conscience, resulted in bringing the child-life of the nation under ever more and more searching scrutiny. The organisation of the school has, in the eyes of philanthropists of all sorts, provided the means for exploiting the children in the interests of a variety of "social reforms," not all necessarily in harmony with the interests of the children themselves. The older notions of school life being devoted to the acquisition of "the three R's," some elementary rules of conduct, and the catechism, have faded away before the new order of things. What education includes or does not include is a problem of some nicety, and we believe we are right in saying that no Education Act has yet vouchsafed a definition of the word. There are those, like the Socialists, who would frankly confide to the State the whole, or nearly the whole, responsibility for the physical, mental, and moral welfare of the child during school age, namely, five to fourteen, and would demur to the artificial limitations which such school age implies. On the other hand, there is scarcely any individualist bold enough to dispute the wisdom of the steps taken by the Legislature in the 'seventies of last century, and ready to advocate the relegation of the education of the child to the parent or to voluntary charity. It is, indeed, not easy, when once the obligation of the State to provide and enforce education is conceded, to draw a line which shall delimit the frontier of parental and State responsibility respectively.

The chain of reasoning runs thus: The State, by compelling the child to attend school, *i.e.* to go to a certain place for a number of hours daily for a number of years, assumes some degree of responsibility for the welfare of the child, and by undertaking to provide what is called education out of moneys extracted from the payers of taxes and rates, is bound to have regard to the quality and quantity of the article supplied. Not only has the State assumed liability to see that the child suffers no detriment in being put through the process of being made into a young citizen, but it may reasonably be expected to secure that the child is able to take the best advantage of the tuition supplied and the training ordained.

Some such argument as this appears to have afforded the basis for the Education (Provision of Meals) Act, 1906, and for the Education (Administration Provisions) Act, 1907. In both cases the orthodox justification of such enactments would appear to be that they provide machinery which is ancillary to education. To teach the underfed is condemned as a labour of Sisyphus; to ignore physical defects and diseases, whose removal or cure would greatly facilitate the educational process, is wasteful as well as foolish. But here, again, are those who are already asking if necessitous children are to be fed at the public expense (in default of voluntary or parental provision) during term time, then why not also during holidays? And if between the ages of five and fourteen, why not from birth to adolescence, even if you stop there? So, also, there are those who say if medical inspection results in showing the necessity for medical treatment, why should the latter not be as much an obligation on the local authority as the former, and why should school age be the artificial span of life exclusively submitted to such benevolent despotism?

We are here clearly beset with problems of a far-reaching character, and the loose phrasing of the Act of 1907 does not help us. By Section 13 of that Act it is obligatory on a local authority to make provision for medical inspection, and it is further enabled to "make arrangements for attending to the health and physical condition of the children." Seldom has a large new departure in legislation been enacted in such lax and ambiguous terminology. Two years later certain doubts were cleared up by the Local Education Authorities (Medical Treatment) Act, 1909, whereby medical treatment was made a charge upon the parent, and at the same time it was made clear that no obligation to submit his child either to medical inspection or treatment rested upon the parent. As might have been anticipated, different local education authorities have taken very different views of the duties imposed upon them and the modes in which these should be carried out, though most argue that since the duties have been put on them by Parliament some portion of the cost should have been forthcoming from moneys voted by Parliament.

In some cases, as at Newcastle, it would appear from reports in the Press that children found to be suffering, on inspection at school, from certain defects, such as errors of refraction, have been referred to hospital in inconvenient numbers, but without any financial arrangement having been entered into with the hospital authorities. This was also the case in London until recently, and great was the outcry at the dislocation of the normal work of the hospitals which ensued. The London County Council has now, however, entered into arrangements with several hospitals whereby payment is made in consideration for such "school cases" as are sent, in a way which it is hoped may be convenient to the hospital staffs. It is too early yet to determine how far this scheme will be satisfactory or adequate. There remains, of course, the question of school clinics, erected, staffed, and maintained by the local authorities out of rates. Such a solution of the problem of medical inspection and treatment as this implies, of course, would involve a heavy charge on the rates, and would inaugurate a new municipal service calculated still further to extrude the general practitioner. We note that almost on the last day of the last Parliament a question was put by Sir William Collins to the President of the Board of Education as to the need for some inquiry by Commission or Departmental Committee into the many problems raised directly and indirectly by this quasi-Socialistic legislation of 1906 and 1907; and we are inclined to think that such investigation by a body commanding general confidence would at the present time be both opportune and enlightening.

